# Mechanistic insights on mogrosides as bifunctional regulators of metabolic reprogramming and immune modulation in tumor microenvironment

**DOI:** 10.1007/s00262-026-04478-w

**Published:** 2026-07-24

**Authors:** Meghna Patial, Rohit Joshi, Jyoti Rajput, Vineet Kumar, Janne Ruokolainen, Kavindra Kumar Kesari, Dhruv Kumar

**Affiliations:** 1 School of Health Sciences and Technology (SoHST), UPES, Dehradun, 248007 India; 2 Division of Biotechnology, CSIR-Institute of Himalayan Bioresource Technology, Palampur, India; 3 Academy of Scientific and Innovative Research (AcSIR), CSIR-HRDC Campus, Ghaziabad, Uttar Pradesh India; 4 Chemistry and Bioprospecting Division, Forest Research Institute, Dehradun, 248006 India; 5 Department of Applied Physics, School of Science, Aalto University, Puumiehenkuja 2, FIN-02150 Espoo, Finland; 6 University Center for Research and Development, Chandigarh University, Mohali, Punjab India; 7 Nanomicroscopy Center, Aalto University, Puumiehenkuja 2, FIN-02150 Espoo, Finland

**Keywords:** Mogrosides, AMPK activation, Tumor microenvironment, Immune modulation, PD-L1, STAT3 signaling, Metabolic reprogramming, Adjuvant therapy

## Abstract

Metabolic reprogramming and immune evasion are interconnected hallmarks of cancer that sustain tumor progression and therapeutic resistance. This review summarizes current mechanistic evidence supporting the therapeutic potential of mogrosides, derived from the fruits of *Siraitia grosvenorii*, as multi-functional agents with dual roles in regulating tumor metabolism and immune responses. Mechanistically, mogrosides are potent AMPK activators that attenuate the Warburg effect, suppress PI3K/AKT/mTOR signaling, and inhibit de novo lipogenesis. Also, mogrosides suppress lactate accumulation within the tumor microenvironment. In parallel, they modulate immune responses through inhibition of STAT3 and NF-κB signaling pathways, resulting in reduced pro-inflammatory cytokine production and decreased PD-L1 expression. Additionally, mogrosides interfere with oncogenic signaling cascades including JAK/STAT3 and MAPK/ERK that regulate proliferation and survival. They further suppress epithelial–mesenchymal transition and matrix metalloproteinase expression, limiting metastatic potential. By simultaneously targeting metabolic plasticity and immune suppression, mogrosides represent promising adjuvant candidates in cancer therapy; however, further clinical investigations are required to validate their translational potential.

**Graphical abstract:**

**Figure:**
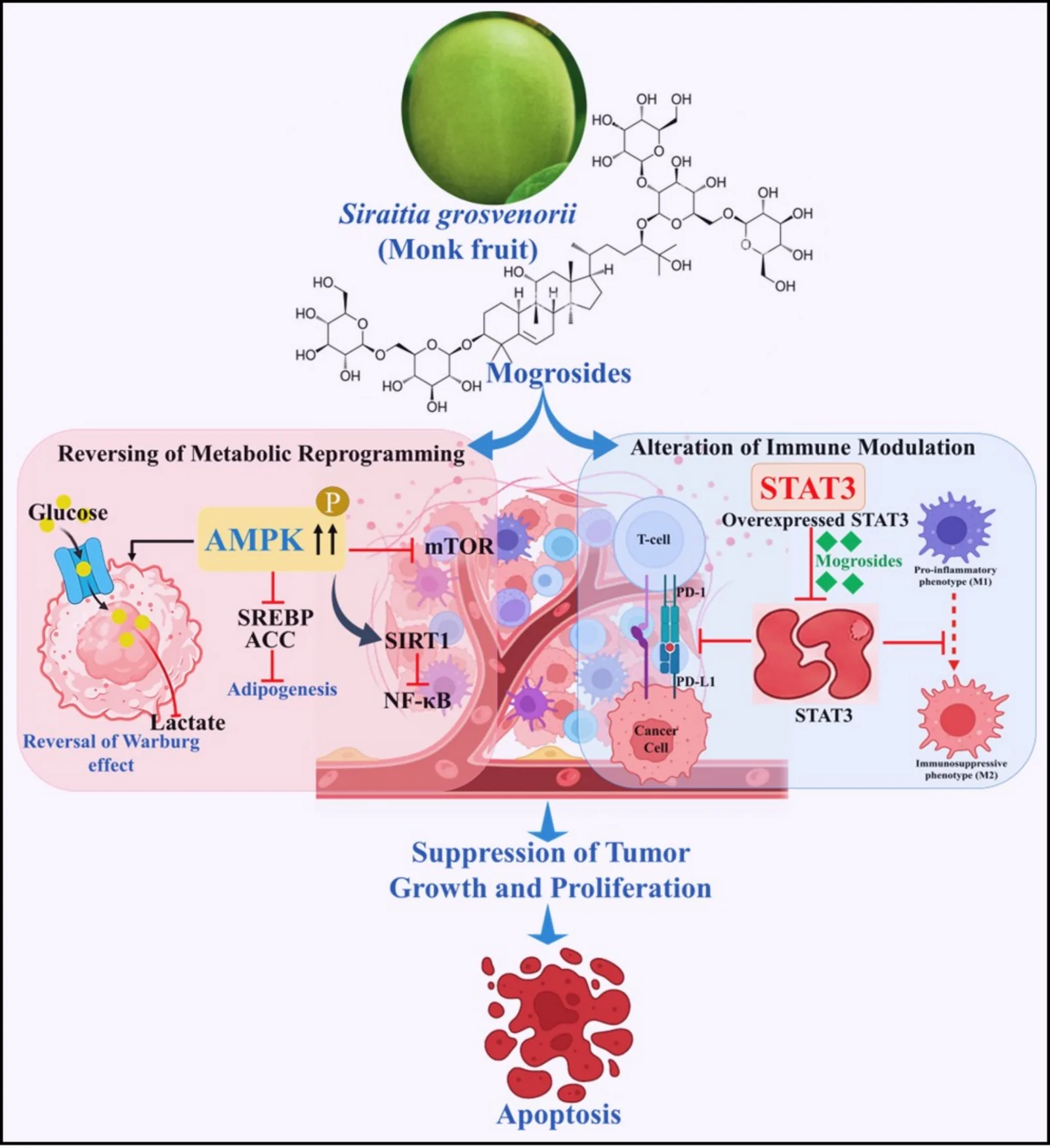

**Supplementary Information:**

The online version contains supplementary material available at https://doi.org/10.1007/s00262-026-04478-w.

## Introduction

Cancer has been the most dreadful disease since the past era. Modern oncological understanding views the tumor not merely as a mass of malignant cells caused by genetic mutations but as a complex systemic pathology driven by the interplay of chronic inflammation, metabolic dysregulation, and dynamic interactions of malignant cells with their microenvironment, also known as Tumor microenvironment (TME) [1]. Metabolic reprogramming and immune evasion have been the interlinked hallmarks of cancer. Central to metabolic reprogramming is the Warburg effect, first described by Otto Warburg, which allows malignant cells to grow and proliferate even under hypoxic conditions [2]. Cancer cells have high metabolic demands for rapid proliferation, which deplete the microenvironment of glucose, amino acids, and oxygen while enriching it with metabolites like lactate. By accumulating such toxic metabolites, and depleting the microenvironment of essential substances like glucose, amino acids, and oxygen, tumor cells create an immunosuppressive environment that impairs the function of cytotoxic T cells [3].

This contemporary perspective of cancer emphasizes the inadequacies of traditional and single-target cytotoxic drugs, which often fail due to tumor heterogeneity. Thus, there is a constant search for effective therapeutic agents that can simultaneously target multiple pathological pathways. Natural products are a rich reservoir of lead compounds that have emerged as promising candidates offering high bio-accessibility, relatively low adverse effects and economic viability. US FDA has approved various phytocompounds like Paclitaxel, Camptothecin and Vincristine for clinical use against various cancers [4].

Among natural products, *Siraitia grosvenorii* (Swingle) C. Jeffrey known as Monk fruit or Luo Han Guo has gained significant attention due to the presence of triterpenoid glycosides called mogrosides. Emerging pharmacological evidence suggests that these compounds possess profound antioxidant and anticancer properties. These glycosides function as pleiotropic agents capable of simultaneously disrupting tumor glycolysis and remodeling the immune microenvironment. Mogrosides provide a holistic anticancer strategy by simultaneously addressing co-morbidities, specifically diabetes, obesity, and chronic inflammation, which are known as systemic drivers of carcinogenesis [5].

## Phytochemistry and ethnobotany of mogrosides

### Chemical constitution of mogrosides

Mogrosides represent a diverse class of cucurbitane-type triterpenoids that share a common tetracyclic aglycone, mogrol. They are differentiated by the number and linkage patterns of their conjugated sugar moieties. Mogrol is a precursor molecule for the synthesis of mogrosides. It is a tetracyclic triterpenoid synthesized through the mevalonate pathway from acetyl-CoA. When the fruit matures, mogrol is glycosylated to mogrosides by UDP-glycosyltransferases. Mogrosides result from epoxidation, hydroxylation, and glycosylation of mogrol [6]. The number of sugar moieties attached to mogrol via β-linkage varies with different developmental stages of fruit, thereby yielding different types of mogrosides [5]. Table 1 lists the different types of mogrosides.

**Table 1:** Different types of mogrosides present in fruits of *S.grossvenorii*

Type of mogroside	Structure	Molecular weight (Da)	Property
Mogrol		476.73	Anticancer, Anti-inflammatory, Antitumor
Mogroside-V		1287.34	Anticancer, Antioxidant, Anti-inflammatory, Anti-diabetic
Isomogroside-V		1287.34	Antioxidant
11-oxo-mogroside**-**V		1285.42	Anti-inflammatory, Antioxidant
Mogroside-IV		1125.29	Antioxidant, Anti-inflammatory
Mogroside-III		963.15	Anti-inflammatory, Neuroprotective, Mild anticancer activity
Mogroside-II		801	Antioxidant, Anti-obese, Hepatoprotective
Mogroside-I		638.9	Antioxidant
Siamenoside-I		1125.29	Anti-inflammatory, Antioxidant
Kaempfertin A		578.5	Antioxidant, Anti-inflammatory, Neuroprotective
Grossvenorine		740.7	Anti-inflammatory, Antitumor, Anti-oxidant

### Ethnobotany of mogrosides

Mogrosides have been in the service of humans for more than 300 years. They are known for their intense sweetness, which is 200–350 times greater than that of sucrose. Their low caloric value and nontoxic nature have led to their widespread adoption in the food and beverage industry. Notably, Mogrosides have been granted the status of GRAS (generally recognized as safe) for their use in dietary supplements by the United States FDA [5]. The traditional Chinese medicine system describes the fruits of *S. grosvenorii* as sweet in taste and cool in nature [7]. Mogrosides have been used as a folk medicine against various ailments like diabetes, obesity, sore cough, dire thirst, constipation, lung congestion, sunstroke, asthma, reduce phlegm, hypertension, and cardiovascular disease [8]. Also, mogrosides have therapeutic properties like antioxidant, anti-diabetic, anti-fibrotic, anti-proliferative, anti-inflammatory, anti-obese, and anti-fatigue [9]. These therapeutic uses of the mogrosides have been listed in the Chinese pharmacopoeia since 1977. Other documents like the National Compendium of Materia Medica, Dictionary of Traditional Chinese Medicine (TCM), Color Atlas of Chinese Herbal Medicine, Chinese Herbal Medicine, and Lingam Materia Medica also highlights the established therapeutic role of mogrosides. Monk fruit has also been recognized as a “Medicine food homology” plant due to its nutritional and therapeutic benefits [5]. However, in the realm of cancer research, while initial studies on cell lines such as prostate, throat, lung, breast, and colorectal are promising, there is currently a lack of scientific evidence for its effectiveness as an anticancer compound.

## Metabolism and bioavailability of mogrosides

**Fig. 1:**
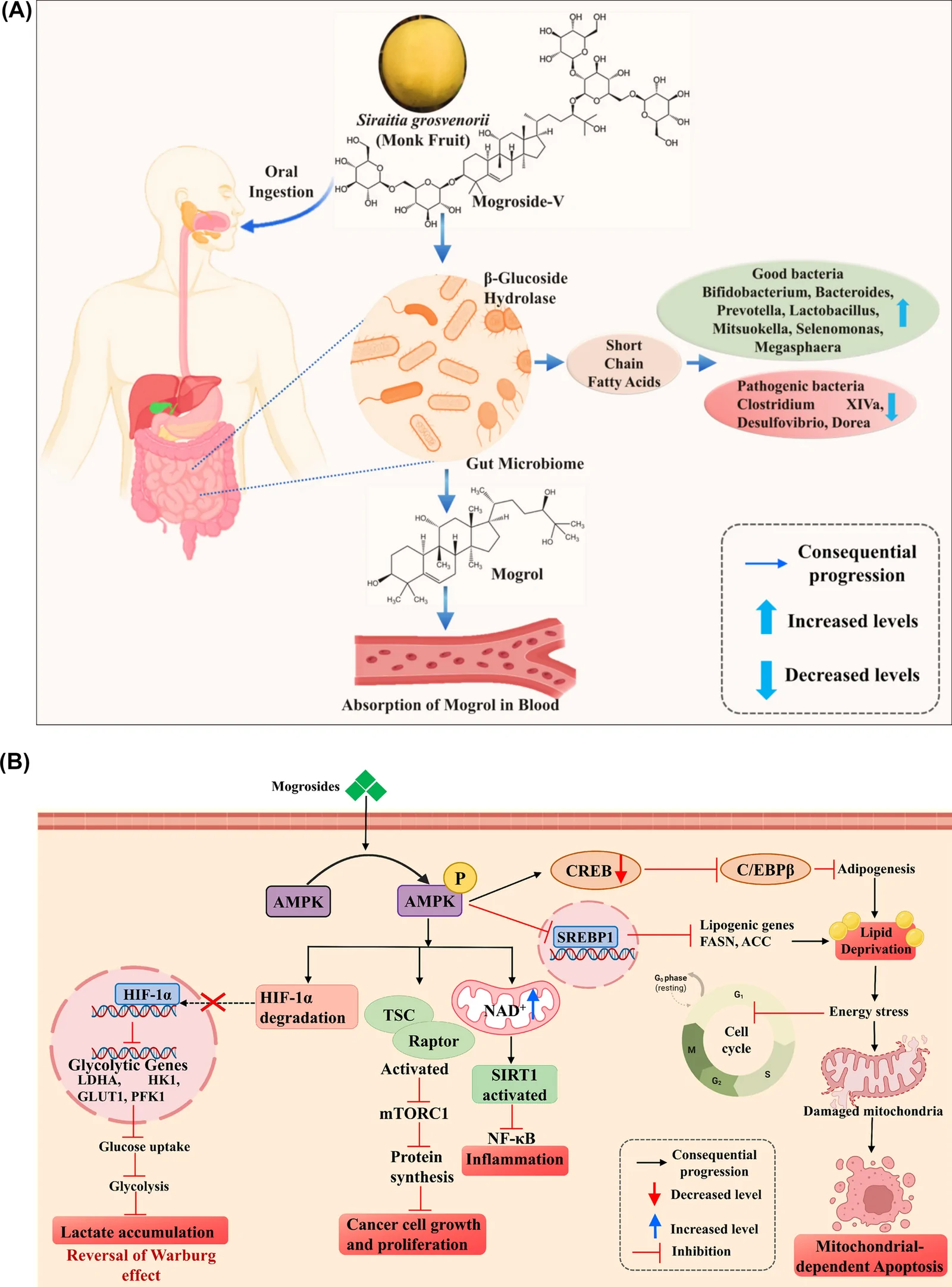
**A** Gut-microbiota mediated metabolism and absorption of mogrosides: β-glucosidase produced by microbiome, primarily in small intestine cleave the sugar moieties present in mogrosides, yielding more bioavailable compound mogrol. Gut microbiome utilizes sugar moieties as an energy source to synthesize short chain fatty acids which promote growth of good bacteria and inhibit pathogenic bacteria. **B** Schematic representation of Metabolic reprograming by Mogrosides via AMPK Signaling Pathway: Mogrosides activate the AMPK pathway which degrades HIF-1α and inhibit mTORC1 which effectively reverses Warburg effect and inhibit protein synthesis. Activation of AMPK also inhibits the transcription of lipogenic genes which deprives cancer cells of lipids required for the synthesis of essential amino acids, leading to cell cycle arrest and mitochondrial-dependent apoptosis

Mogrosides follow complex metabolic transformation in the animal or human body, followed by oral ingestion. Mogrosides, particularly mogroside-V, are not directly absorbed in the small intestine due to their large structure. They undergo gastrointestinal hydrolysis, microbial transformation, absorption and excretion. The acidic conditions in the stomach do not have an effect on mogrosides due to their glycosidic structure. In the small intestine, they exhibit minimal absorption, and hence, they are passed to the large intestine where microbial enzymes such as β-glucosidase hydrolyze the sugar moieties, converting mogrosides into mogrol and some intermediate metabolites. Mogrol has better intestinal permeability and can be absorbed across the gut epithelium into systemic circulation [10]. Figure 1A illustrates the metabolism of mogrosides in the small intestine, primarily by β- glucosidases produced by the gut microbiome. Xu et al. [11] confirmed the presence of mogrol in plasma. In this pharmacokinetic study, it was found that after oral administration of mogrosides, mogrol and mogroside-I were present in the plasma. So, it might be possible that mogrosides are first hydrolyzed extracellularly and then the active molecule enters the cell membrane passively and acts in the cytoplasm [11]. Murata et al. [10] also studied the digestion and absorption of crude mogrosides in vivo models. They quantified the different mogrosides from small intestines and feces, however, only mogroside -I and mogrol were detected in portal blood. It was concluded that mogrosides were mostly degraded by intestinal microflora and excreted in feces. They also reported that only mogrol and its mono substitutes are absorbed in the blood [10]. It has been reported that mogrol (the active molecular structure of mogrosides) is absorbed in animal models and from the above studies, it can be inferred that mogrol is responsible for the curative activities of monk fruit/ mogrosides. Studies on in vitro and in vivo metabolism of mogroside-V have shown that it undergoes a series of reactions like deglycosylation, dehydrogenation, hydroxylation, deoxidation, isomerization, glycosylation, oxidation and methylation to form its active metabolites [12]. Xu et al. [11] conducted the in vitro and in vivo study to investigate the metabolism of mogroside-V. They found that mogroside-V was bio-transformed by human intestinal bacteria and nearly 77 sub metabolites of mogroside-V were detected. These metabolites were randomly distributed in plasma, heart, spleen, stomach, kidney, liver, small intestine, and lungs of in vivo models [11]. Another in vivo study was conducted to determine the metabolite profile of mogroside-V in healthy and type 2 diabetic (T2D) rat models. Results indicats that mogroside-V was metabolically transformed into its metabolites and 23 sub-metabolites were observed in healthy rats, whereas 26 sub-metabolites were detected in T2D rats. The metabolites were observed in plasma, bile, urine and feces. It was concluded from the study that high metabolites in the blood of T2D rats might be the reason for the curative property of mogroside-V against type 2 diabetes [12]. In vivo studies confirm that mogrosides themselves have very low bioavailability, but their aglycon metabolite, mogrol, has significantly higher bioavailability.

## Mogrosides in attenuating oncogenic metabolic reprogramming

Cancer cells are metabolically hyperactive, which enables them to rely on a process known as the Warburg effect, which supports their continuous growth, rapid proliferation, and survival even in hypoxic conditions. This is characterized by aerobic glycolysis, de novo lipogenesis and glutaminolysis. Mogrosides, particularly mogrol and mogroside-V act as potent metabolic reprogrammers by intervening in these dysregulated pathways and restoring metabolic homeostasis as illustrated in Fig. 1B.

### Activation of AMPK signaling

Glucose is the main source of energy for cancer cells. The pivotal mechanism by which mogrosides disrupt the oncogenic metabolism is the activation of AMPK (Adenosine Monophosphate-Activated Protein Kinase), a cellular energy sensor and tumor suppressor. Mogrosides, particularly Mogrol, upregulate the phosphorylation of AMPK, which promotes energy homeostasis and reverses the metabolic reprogramming in cancer cells, thereby inhibiting their growth and sensitizing them to apoptosis [13]. Activation of AMPK leads to phosphorylation of TSC2 and Raptor, which inhibit mTORC1 signaling, thereby causing inhibition of lipid and protein synthesis required for cancer cell growth [14]. Increased levels of AMPK also lead to an increase in NAD ^+ ^levels, which supports the activation of SIRT1, thereby inhibiting NF-κB pathway [15]. During aerobic glycolysis (Warburg effect), HIF-1α is stable even in the presence of oxygen. Activation of AMPK degrades HIF-1α, which prevents its accumulation in the nucleus, hence cells cannot transcribe the genes such as GLUT1, HEK2, LDHA for the glycolytic pathway [16].

### Lipid metabolic rewiring and lipotoxicity

Cancer cells require large amounts of lipid molecules for the synthesis of cell membranes and signaling molecules. They significantly upregulate de novo lipogenesis controlled by key lipogenic enzymes such as Fatty Acid Synthase (FASN) and Acetyl-CoA Carboxylase (ACC) [17]. Mogrosides, particularly mogroside-V modulate lipid metabolism through the AMPK-SREBP axis. Mogroside-V phosphorylates and activates AMPK, the master regulator of metabolism. The activation of AMPK causes the downregulation of SREBP (Sterol Regulatory Element-Binding Protein 1), which is a transcription factor for lipogenic gene expression [18]. Activation of AMPK also inhibits the phosphorylation ACC. Concurrently, when phosphorylation of AMPK is upregulated, the action of CREB (cAMP response element binding protein) is downregulated [19]. Suppression of CREB inhibits C/EBPβ (CCAAT/enhancer-binding protein β), the main regulator of adipogenesis, which effectively halts the accumulation of triglycerides and forces cancer cells to shift from catabolic utilization of glucose and lipids [20]. Consequently, tumor cells are deprived of the essential lipids required for cell membrane synthesis and oncogenic signaling. This leads to energetic stress, forcing cancer cells to ultimately undergo G0/G1 cell cycle arrest and mitochondrial-dependent apoptosis.

### Insulin and glucose homeostasis

Dysregulated glucose metabolism and hyperinsulinemia are deeply associated with an enhanced risk of cancer. Excessive circulating glucose acts as a substrate for the Warburg effect. This chronic hyperglycaemia forces pancreatic cells to secrete excess insulin (hyperinsulinemia). Insulin and IGF-1 (Insulin Growth Factor-1) bind to tyrosine receptors on cancer cells, leading to hyperactivation of PI3K/Akt/mTOR and Ras/MAPK pathways, which are associated with excessive proliferation of cancer cells and inhibition of apoptosis [21]. Concurrently, under chronic hyperglycaemia, glucose reacts with proteins to form Advanced Glycation End Products (AGEs). AGEs bind to their receptors on tumor cells and trigger the release of pro-inflammatory cytokines like IL-6, TNF-α via NF-kB pathway, thereby creating a chronic inflammatory microenvironment necessary for tumor progression [22]. Mogrosides act as metabolic interceptors and disrupt this specific loop by enhancing insulin sensitivity and glucose uptake by normal tissues, which lowers the circulating glucose. This directly reduces the availability of glucose for the Warburg effect as well as for the formation of AGEs, which trigger the formation of pro-inflammatory cytokines [23]. Mogrosides are natural antioxidants that help neutralize ROS and other inflammatory cytokines. Furthermore, mogrosides directly inhibit the NF- kB pathway, which is necessary for the transcription of pro-inflammatory cytokines. Hence, mogrosides interfere with hyperglycaemia-induced AGE and cytokine cascade at multiple levels, thereby disrupting the conditions necessary for cancer cells survival and growth.

## Mogrosides in immune modulation: reprogramming the tumor microenvironment

**Fig. 2:**
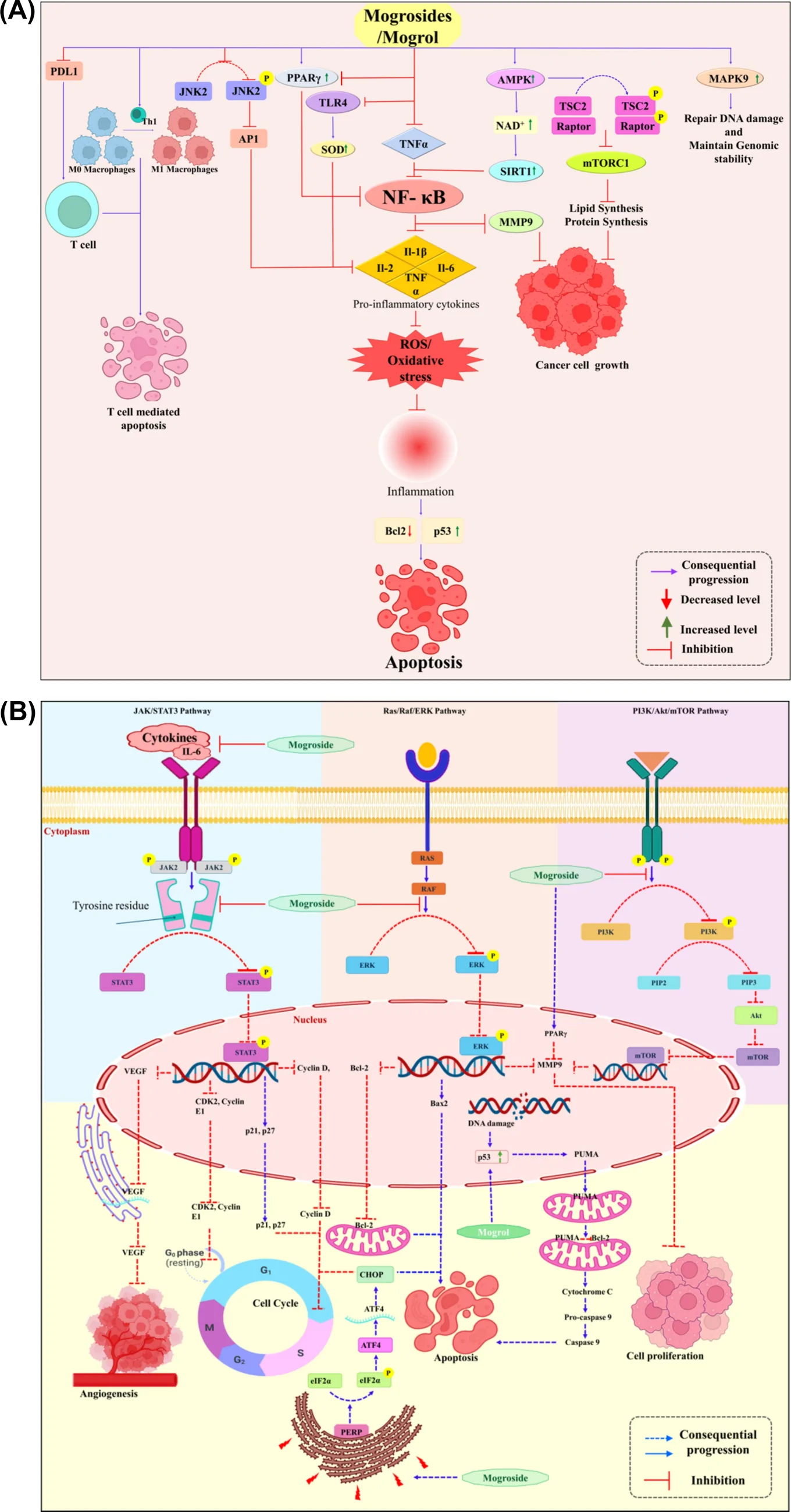
**A** Schematic representation of Antitumor activity of Mogrosides/Mogrol driven by immune modulation within Tumor Microenvironment: Mogrosides and Mogrol drive antitumor activity by facilitating immune modulation through PD-L1 inhibition and M1 macrophage polarization, which restores T cell-mediated apoptosis. They simultaneously remodel the tumor microenvironment (TME) by suppressing the NF-κB inflammatory axis and the mTORC1 growth pathway via AMPK activation. These collective actions reduce pro-inflammatory cytokines and oxidative stress, shifting the TME toward a state that triggers p53-dependent apoptosis. **B** Schematic representation of Regulation of Multiple Oncogenic Pathways by Mogrosides to inhibit Carcinogenesis. The figure illustrates the inhibitory effects of Mogrosides/Mogrol on three major pro-proliferative signaling cascades: the JAK/STAT3, Ras/Raf/ERK, and PI3K/Akt/mTOR pathways. This modulation leads to G1 cell cycle arrest, decreased angiogenesis (through VEGF suppression), and the induction of apoptosis via both intrinsic and extrinsic pathways, ultimately suppressing malignant cell growth

The anticancer potential of mogrosides also stems from their ability to modulate the immune system, particularly within the tumor microenvironment, where immune suppression is a major hallmark that allows cancer cells to evade detection. Mogrosides not only modulate the immune suppression within TME but also restructure the TME to favor apoptosis of cancer cells, as illustrated in Fig. 2A.

### Macrophage polarization

Tumor-associated macrophages are the major constituents of TME, which either adopt an antitumor, pro-inflammatory M1 phenotype or a pro-tumor, immunosuppressive M2 phenotype. In the majority of tumors, the M2 phenotype predominates, which secretes anti-inflammatory cytokines such as IL-10, TGF-β and promotes angiogenesis [24]. Mogrosides inhibit the STAT3 pathway, which prevents the transcription of M2 genes required to maintain the pro-tumor M2 phenotype. Mogrosides also increase the phagocytic capacity of macrophages, which are essential immune cells that engulf pathogens and release cytokines to recruit other immune cells. Although M1 macrophage is known to exert the antitumor effects, however, its chronic activation leads to sustained inflammation and tissue damage within the TME. Notably, Mogroside-V has been reported to suppress the excessive M1 polarization via p38 MAPK/NF- κB signaling pathway which reduces the overproduction of inflammatory mediators under pathological inflammatory conditions characterized by dysregulated macrophage activation [25]. Concurrently, mogrosides also modulate the lactate production in tumors, which removes the key signal that drives M2 polarization [26].

### Targeting PD-1/PD-L1 (programmed cell death protein-1/programmed death-ligand 1) axis

PD-1/PD-L1 axis acts as a critical immune checkpoint that helps cancer cells to evade the immune system. Under normal physiological conditions, PD-1, present on the surface of T cells, prevents overactivation of the immune system to avoid attacking healthy cells (autoimmunity). However, cancer cells exploit this mechanism by overexpressing PD-L1 on their own surface. PD-L1 is a major immune checkpoint protein that binds to PD-1 and enables cancer cells to evade immune surveillance by inducing T cell exhaustion. High PD-L1 expression in tumors is driven by the continuous activation of STAT3 [27]. PD-L1 is encoded by the *CD274 *gene, which is a direct transcriptional target of STAT3. Mogrosides, particularly, Mogroside-V and Mogrol act as a potent inhibitor of STAT3, which in turn suppresses the expression of PD-L1, which in turn enhances T cell-mediated tumor destruction, representing a potent and clinically relevant immune-modulatory mechanism [28, 29].

### Cytokine modulation and resolving chronic inflammation

Inflammation is a complex biological response of the immune system triggered by harmful stimuli such as toxins, pathogens, and damaged cells. The response involves the release of various chemokines, ROS, pro-inflammatory cytokines (such as TNF-α; IL-1β,2,6), and prostaglandins. The fundamental driver of tumor pathogenesis is the chronic inflammation, which is characterized by the continuous activation of the immune system and elevated levels of pro-inflammatory cytokines [30]. Tumor cells exploit PI3K/Akt and NF-κB pathways to sustain a pro-inflammatory microenvironment. The PI3K/Akt pathway activates Akt which increases ROS levels and contributes toward inflammation by triggering the NF- κB pathway [31]. Mogrosides, particularly Mogroside-V and 11-oxo-mogrol, effectively attenuate the inflammatory signal by inhibiting the PI3K/Akt pathway. Mogroside-V also contributes to the resolution of inflammation by downregulating the expression of critical pro-inflammatory proteins, including the activated NF-κB subunit p-P65, cyclooxygenase-2 (COX-2), and inducible nitric oxide synthase (iNOS) [32]. Additionally, Mogrol can also suppress NF-kB pathway, which is associated with various chronic inflammations such as cancer, neuroinflammatory diseases, inflammatory bowel diseases, and rheumatoid arthritis [33]. Mogroside-V also inhibits the activation or expression of NF-κB, activator protein1/heme oxygenase-1 and human CAAT-enhancer-binding protein delta (C/EBPδ), thereby suppressing the inflammation. Furthermore, Mogroside -IIIE and Mogroside-V also combat inflammation by activating AMPK, which in turn downregulates TLR4/MAPK/NF- κB signaling pathway. They upregulate the AMPK/SIRT1 signaling pathway and inhibit the inflammatory cytokines (TNF- α and IL-2,6), which results in downregulation of oxidative stress-related markers [34]. Moreover, mogroside induced SIRT1 activation also leads to suppression of inflammation via downregulation of NF-κB as SIRT1 is a negative regulator of NF-κB [35].

Collectively, the ability of mogrosides to regulate pro-inflammatory cytokines and oxidative stress highlights their role in resolving chronic inflammation. Beyond these effects, emerging evidence suggests that mogrosides may exert broader immunomodulatory functions extending into adaptive and regulatory immune compartments. Mogrosides modulate the key signaling pathways such as TLR4/MAPK/NF-κB and AMPK/Nrf2 that influence immune cell activation and differentiation [36]. These mechanisms further suggest the potential role of mogrosides in enhancing T cell-mediated immunity, including Th1 polarization and CD8⁺ cytotoxic responses, as well as supporting B cell function and antibody production. Furthermore, regulation of cytokines and redox may favor improved natural killer (NK) cell activity and immune surveillance. Suppression of chronic inflammatory signals on the other hand would reduce populations such as Tregs, Bregs, and MDSCs that dampen immune responses, while also potentially functioning to inhibit N2 neutrophil polarization and Th2 skewed responses [37]. While these mechanisms have not been examined directly in the present study, they are mechanistically plausible and require further investigation.

## Modulation of oncogenic signaling pathway

Mogrosides exert their effects by directly modulating a complex network of pivotal signaling pathways that are often dysregulated in cancer, as shown in Fig. 2B.

### JAK2/STAT3 pathway inhibition

In normal cells, cytokines or growth factors bind to their specific cell receptors, particularly associated with JAK2. The binding of these receptors with ligands auto-phosphorylates and activates JAK2. Activated JAK2 further phosphorylates STAT3 on a key tyrosine residue (Tyr 705). Phosphorylated STAT3 is then translocated to the nucleus, where it binds to specific DNA sequences and regulates cell proliferation, differentiation, immune response, and apoptosis. However, continuous activation of STAT3 leads to uncontrolled proliferation, angiogenesis, immune evasion, resistance to apoptosis, and metastasis in cancer cells [38]. Mogrosides block the phosphorylation of STAT3 at Tyr705 residue. This prevents the translocation of STAT3 into the nucleus. Consequently, the transcription of genes like *Bcl*, *CDK2*, *Cyclin D1* and *Cyclin E* is also suppressed, thereby promoting apoptosis and inducing cell cycle arrest at G0/G1 phase [39]. Furthermore, by inhibiting STAT3 activation, mogroside-V also downregulates the transcription of the VEGF gene responsible for the translation of the VEGF protein, responsible for angiogenesis, providing nutrients and oxygen to tumor cells [40]. In addition, mogrosides also restore the negative feedback loop by enhancing the expression of SOCS3 (Suppressor of cytokine signaling), a natural inhibitor of JAK2 [41]. Mogrosides also downregulate the expression of IL-6, a cytokine which is known to activate JAK/STAT3 pathway in various cancers, including breast, ovarian, and pancreatic [42].

### MAPK/ERK pathway regulation

Mogroside-IVe and its aglycon mogrol have been shown to suppress the phosphorylation of extracellular signal-regulated kinases (p-ERK1/2) [13]. This pathway plays a crucial role in the survival and progression of cancer cells. Genetic mutations or overexpression of upstream components, such as ERK, foster oncogenic transcription factors such as cyclin D1. By inhibiting the phosphorylation of ERK1/2, mogrosides prevent its nuclear translocation and transcriptional activity. Consequently, the expression of Cyclin D1 is downregulated, which leads to cell cycle arrest at G1/S phase. Continuous activation of ERK1/2 also induces the transcription of bcl-2, which promotes cell survival and inhibits pro-apoptotic genes [43]. By inhibiting the phosphorylation of Erk1/2, mogrosides also control the expression of the *Bcl-2* gene, thereby leading to apoptosis [9].

### PI3K/Akt/mTOR pathway inhibition

This pathway plays a major role in cancer cell survival, proliferation, and inhibition of apoptosis. Under normal physiological conditions, this pathway maintains cellular homeostasis. In many cancers, it becomes hyperactivated, leading to uncontrolled tumor growth. Mogrosides intervene in this pathway by inhibiting the phosphorylation of PI3K, which suppresses its catalytic activity of converting membrane phospholipid PIP2 into PIP3 [44]. Depletion in PIP3 levels leads to the removal of essential membrane docking sites required for the activation of Akt, the key player of the PI3k/Akt/mTOR pathway [45]. Consequently, Akt remains in inactive form in the cytosol and cannot trigger its downstream cascade, which also involves the activation of mTOR, the nutrient sensor and protein manager of cells. In cancer cells, when Akt is hyperactivated, it inactivates TSC1/2, which allows the activation of Rheb and subsequently of mTORC1 [46]. By maintaining Akt in its inactive form, mogrosides allow for the activation of TSC1/2 and inhibition of Rheb, which suppresses the activity of mTORC1, which effectively obstructs the protein synthesis required for G1 to S phase transition, thereby arresting the cell cycle.

### Induction of ER stress and apoptosis

A crucial and specific mechanism of mogrosides' anticancer action is their ability to trigger endoplasmic reticulum (ER) stress, a condition marked by the accumulation of misfolded proteins. Mogrosides induce ER stress, which in turn activates PERK, the ER stress sensor [7]. Activated PERK phosphorylates eIF2α, which reduces the overall protein synthesis. However, it increases the synthesis of ATF4 protein, which enters the nucleus from the cytoplasm and binds with specific DNA sequences and initiates the transcription of CHOP mRNA to synthesize CHOP protein. CHOP protein is a key factor that promotes apoptosis during ER stress [47]. The main regulator of apoptosis and senescence is p53, also known as the guardian of the genome. It is a major tumor suppression protein, and its functional inactivation is common in many cancers. Mogrosides enhance the activation and expression of p53, thereby facilitating apoptosis and cell cycle checkpoint control [13]. Elevated levels of p53 upregulate pro-apoptotic proteins such as PUMA, which inhibit the expression of anti-apoptotic genes such as Bcl-2. This results in the release of cytochrome c in the cytoplasm from mitochondria, cytochrome c further activates caspase 9, which triggers apoptosis [48].

### Anti-metastatic effects

Mogroside-V has been shown to inhibit cancer cell migration and invasion by reversing the process of Epithelial–Mesenchymal Transition (EMT) [28]. EMT is a reversible process by which immotile epithelial cells acquire a mesenchymal phenotype through biochemical and morphological changes. This is characterized by invasiveness, migratory capacity, and resistance to apoptosis, leading to metastasis of cancer cells. The “Cadherin switch” is a definitive hallmark of EMT controlled by loss of E-cadherin, gain of N-cadherin and expression of Vimentin [49]. Mogrosides achieve the anti-metastatic effect by functioning as biochemical modulators that restore the expression of E-Cadherin, a key epithelial marker, while downregulating mesenchymal markers such as N-Cadherin, Vimentin, and Snail. Furthermore, mogrosides particularly mogroside-V been found to break the microfilaments that form the cellular cytoskeleton, which reduces the expression of key Rho-GTPase family proteins such as Rho A, Rac1, and Cdc42 that help cancer cells to migrate and invade [28]. Additionally, mogrosides significantly suppress matrix metalloproteinases (MMPs), particularly MMP9, which plays a key role in degrading the extracellular matrix and promoting cell invasion [50]. The expression of MMP9 is regulated by the ERK and NF-κB pathways, both of which are downregulated by mogrosides. MMP9 is also a key target of PPARs, particularly of PPARγ. PPARγ is a nuclear receptor involved in adipogenesis, glucose metabolism, and inflammation. In cancer cells, its activation promotes differentiation, reduces proliferation, and inhibits metastasis. Mogrosides activate PPARγ, resulting in suppression of oncogenic pathways and induction of cell cycle arrest [51].

### Anti-angiogenic effects

Angiogenesis is a process by which cancer cells form new blood vessels to supply the tumor with nutrients and oxygen [52]. Research findings suggest that mogroside-V significantly reduces the expression of the pro-angiogenic factor VEGF, which stimulates the formation of new blood vessels. By decreasing VEGF levels, mogrosides-V effectively starves the tumor cells of the resources they need to grow [9]. The anti-angiogenic effects are believed to be mediated, at least in part, by the inhibition of the STAT3 pathway, which is an essential mediator of VEGF transcription [52].

## Clinical implications and future prospects

### Preclinical evidence in various cancer types

The preclinical evidence for the anticancer effects of mogrosides is compelling and spans a wide range of cancer types. In silico docking studies have identified specific protein targets, including interleukins, growth factors, and kinases, confirming their targeted mechanism of action.  In vitro and in vivo studies have demonstrated the efficacy of mogrosides against pancreatic, lung, colorectal, laryngeal, prostate, bladder, breast, and liver cancers, as well as leukemia, as referred to Table 2. Additionally, in vitro findings have been successfully validated in xenograft mouse models, which confirms that Mogroside-V can inhibit tumor growth in living organisms.

**Table 2:** Anticancer studies of mogrosides against various cancer cell models

S. No	Compound	Cancer cell lines	Animal model	References
1.	Mogroside-V, IV, III	Skin cancer (TPA induced iNOS)	Female ICR mice	[53]
2.	Mogrol	K-562 (Leukemia)	–	[54]
3.	Mogrol	CNE1 and CNE2 (nasopharyngeal), DU145 (Prostate), HepG2 (Liver) and A549 (Lung)	–	[50]
4.	Mogroside-V	A-549 and H1299 (Lung)	–	[55]
5.	LLE and MOG (commercial products)	T24 (Bladder), PC-3 (Prostate), A549 (Lung), MD-MB231 (Breast) and HepG2 (Liver)	–	[56]
6.	Mogrol	Non-small-cell lung carcinoma cells (A549, H1299, H1975 and SK-MES-1 cells)	Male thymus deficient mice (BALB/C)	[13]
7.	*Siraitia grosvenorii* Polysaccharide (SGP-1)	MDA-MB-231 (Breast), HepG2 (Hepatoma) and A549 (non-small cell lung cancer cell line)	–	[55]
8.	LLE and MOG (commercial products)	PC-3 (Prostate) and T-24 (Bladder)	–	[57]

### Safety profile of mogrosides

The safety profile of mogrosides is robust, which establishes a strong foundation for their consideration as a therapeutic candidate. Their safety has been verified by various health agencies around the globe, including Food Standards Australia New Zealand, Health Canada, Japan’s Ministry of Health, Labor and Welfare and no adverse effects have been reported in humans, including for pregnant women, breastfeeding women, or infants. Animal studies have further strengthened this safety profile. A median lethal dose (LD50) of > 15 g/kg suggests that mogrosides are nontoxic even at high doses. This significantly higher tolerance level is more than that of common food substances like table salt (LD50 of 3 g/kg) and citric acid (LD50 of 6.73 g/kg) [58]. Furthermore, a 90-day study on male and female dogs administered a high dose of 3000 mg/kg body weight/day recorded no adverse effects in any clinical measure, including body weight, blood chemistry, or histopathology [59]. The no observed adverse effect level (NOAEL) of dietary mogrosides was determined to be 7.07 g/kg body weight/day for male rats and 7.48 g/kg body weight/day for female rats, demonstrating exceptional tolerability [60]. An Ames mutagenicity test also revealed that mogrosides do not cause direct or indirect mutations. The proposed levels of mogroside-V for usage vary according to the food category, like 800 mg/Kg for weight control diets, 1000 mg/Kg for dietary foods (medical purpose), 3200 mg/Kg for tabletop sweeteners, and 5500 mg/Kg for Food supplements. Studies on reproductive and developmental toxicity have been largely reassuring. A reproductive and developmental screening study in rats, involving daily doses of up to 4,000 mg/kg body weight/day, found no adverse clinical or reproductive effects on the parental generation or on the development of their offspring. Specifically, no maternal or developmental toxicity was observed in a prenatal study at a dose of 1,000 mg mogroside -V/kg body weight/day [61]. Further, Monk fruit extracts are well tolerated in humans at a dose of 0.51g/day without affecting postprandial glucose levels or any other significant adverse effects [62].A comprehensive safety profile of mogrosides is shown in Supplementary Table 1. This extensive safety data provide a crucial advantage for translational research. Unlike a novel synthetic compound that would require extensive and risky Phase I trials, mogrosides can likely move to proof-of-concept and efficacy trials more quickly, presenting a unique and accelerated path for drug development**.**

### Adjuvant potential of mogrosides

The most immediate clinical application of mogrosides is their ability to enhance the efficacy of existing drugs while protecting normal tissues from toxicity.

Cisplatin: Cisplatin is a platinum-based chemotherapy used for various types of cancers such as lung, bladder, cervical, and ovarian cancers. However, its common side effects include nephrotoxicity and the development of resistance. When cisplatin is combined with mogrosides, the apoptotic threshold is lowered as mogrosides downregulate *Bcl-2* and survivin, proteins that mediate cisplatin resistance. Also, mogrosides protect renal tubular cells from the cisplatin-mediated oxidative damage.

Doxorubicin: It is a chemotherapy medicine used to treat various cancers such as breast, bladder, and leukemia. Co-treatment of doxorubicin with mogrosides enhanced apoptosis in breast and lung cancer models. The major side effect of doxorubicin is cardiotoxicity caused by ROS generation. Mogroside-V boosts endogenous antioxidant enzymes such as SOD and catalase and activates the Nrf2 pathway, which protects cardiac function during doxorubicin therapy [63].

## Conclusion

The transition of mogrosides from natural non-caloric sweeteners to potential anticancer agents reflects an emerging area of translational interest in oncological research. As summarized in this review, mogrosides, particularly Mogroside-V and its aglycone metabolite Mogrol, exert pleiotropic activity that targets two interrelated hallmarks of cancer: metabolic reprogramming and immune evasion. Unlike conventional chemotherapeutics that frequently act through a single molecular pathway, mogrosides restore metabolic homeostasis through activation of AMPK, thereby counteracting the Warburg effect, inhibiting de novo lipogenesis, and suppressing the PI3K/Akt/mTOR signaling axis. These metabolic effects also contribute to the remodeling of the tumor microenvironment. Mogrosides reduce lactate accumulation, suppress STAT3/NF-κB signaling, reduce M2 macrophage polarization, and downregulate PD-L1 expression, thereby helping to restore antitumor immune surveillance. Furthermore, mogrosides induce ER-stress-mediated apoptosis, enhance p53 activity, inhibit EMT, and downregulate MMPs, collectively limiting tumor progression and metastatic potential of cancer cells. The significant advantage of mogrosides is their high experimental tolerability (LD50 > 15 g/kg) when compared to other chemotherapeutic drugs. This provides a major support to their use as adjuvant agents, which could improve the efficacy of traditional chemotherapeutic drugs while simultaneously protecting the healthy cells from drug-induced toxicity. Overall, the accumulating research suggests mogrosides as promising multi-targeted modulators of tumor metabolism and immunity with significant translational potential in cancer prevention and therapy.

## Future prospects

Mogrosides have strong therapeutic potential, but there are several challenges and opportunities that could be addressed to translate mogrosides into clinical applications. The major limitation of mogrosides is their low systemic bioavailability due to degradation by the gut microbiome. Future research must focus on developing advanced drug delivery systems, such as nano-formulations (liposomes, polymeric nanoparticles) or structural modifications, to protect mogroside-V from deglycosylation in the intestine and enhance the delivery to target tissues. The anticancer efficacy of mogrosides has been validated by in vitro and in vivo xenograft models but human clinical trials are noticeably absent. Randomized controlled trials (RCTs) are needed to determine the effective therapeutic dosage in humans and to confirm the chemopreventive and adjuvant efficacy as observed in animal models. Furthermore, mogrosides help in mitigating the side effects caused by chemosynthetic drugs, so future investigations can also focus on the robust combination therapies. In addition, future studies can be done to investigate whether mogrosides can modulate adaptive immune responses including T and B cell activation, enhance NK cell function, and suppress immunoregulatory populations such as Tregs, MDSCs, and N2 neutrophils, which would strengthen their therapeutic potential. With optimized delivery systems and well-designed clinical trials, mogrosides hold significant potential to enhance current cancer treatment paradigms and improve the quality of life for cancer patients.

## Supplementary Information

Below is the link to the electronic supplementary material.

Supplementary file1 (DOCX 22 kb)

## Data Availability

No datasets were generated or analysed during the current study.

## Abbreviations

ADI: Acceptable daily intake
AGE: Advanced glycation end products
AMPK: 5' AMP-activated protein kinase
ATF4: Activating transcription factor-4
ATP: Adenosine triphosphate
AR: Androgen receptor
Bcl-2: B cell lymphoma
CAT: Catalase
CCAAT: Cytosine–cytosine adenosine thymidine
CCL4: Chemokine ligands 4
CDK: Cyclin dependent kinase
CHOP: C/EBP homologous protein
CHK: Checkpoint
COX: Cyclooxygenase
CREB: CAMP response element binding protein
C/ EBPβ: CCAAT/enhancer-binding protein β
DMBA: 7,12-Dimethyl benzene[a]anthracene
ERK1/2: Extracellular signal-related kinases 1 and 2
GRP78: Glucose Regulated protein 78
GSH-Px: Glutathione peroxidase
HIF-1α: Hypoxia inducible factor 1 subunit α
HSP90AA1: Heat shock protein 90α
HUVEC: Human umbilical vein endothelial cells
IGF-1: Insulin- like growth factor 1
IL: Interleukin
IҠK: I kappa B kinase
JAK: Janus kinase
JNK: C-Jun N-terminal kinase
LDL: Low density lipoprotein
LPS: Lipopolysaccharide
MAPK/MEK: Mitogen activated protein kinase
MDA: Malondialdehyde
MMP9: Matrix metallopeptidase 9
mTOR: Mammalian target of rapamycin
MyD88: Myeloid differentiation primary response protein
NAFLD: Non-alcoholic fatty liver disease
NLRP3: NOD-, LRR-, and pyrin domain-containing protein 3
NF: ҠB- nuclear factor- kappa B
NO: Nitric oxide
iNOS: Nitric oxide synthases
PGC-1α: Peroxisome proliferator-activated receptor-gamma coactivator
PI3K: Phosphoinositide 3-kinase
PLK: Polo-like kinases
PPAR: Peroxisome proliferator-activated receptor
p-RB: Retinoblastoma protein
PRKCA: Protein kinase C α
PRKCB: Protein kinase C β
PUMA: P53 up-regulated modulator of apoptosis
ROS: Reactive oxygen species
SCFAs: Short chain fatty acids
SGP: *Siraitia grosvenorii* Polysaccharide
SIRT1: Sirtuin 1
SOD: Superoxide dismutase
SpPRY1: Sprouty RTK signaling antagonist 1
SQSTM: Sequestosome
STAT: Signal transducer
STAT3: Signal transducer and activator of transcription 3
TFAM: Transcriptional factor A, mitochondrial
TGF: Transcriptional growth factor
TGR: Takeda G-protein receptor
TLR: Toll like receptor
TME: Tumor microenvironment
TNF-α: Tumor necrosis factor
