# Supplementary material for: Mechanistic insights on mogrosides as bifunctional regulators of metabolic reprogramming and immune modulation in tumor microenvironment
Source: Cancer Immunol Immunother. 2026 Jul 24;75(11):256. doi: 10.1007/s00262-026-04478-w (PMC13710497; doi:10.1007/s00262-026-04478-w)
Supplement: Supplementary file 1 [file 262_2026_4478_MOESM1_ESM.docx]

**Table 3: Assessment of safety profile of mogrosides across different models**

| **Compound tested** | **Model** | **Study type** | **Dosage Tested** | **Optimized Dosage** | **Key findings** | **References** |
| --- | --- | --- | --- | --- | --- | --- |
| Monk fruit Extract (40% Mogroside-V) | Male and Female Rats | Subchronic Toxicity | Up to 1,200 mg/kg body weight/day | NOAEL was 7.07 g/kg bw/day for males and 7.48 g/kg bw/day for females. | Generally well-tolerated. No adverse changes, such as increased relative organ weights (liver, adrenal, kidney) in rats at high doses. However, these changes were often not considered toxicologically significant. | ^62^ |
| Mogroside-V | Rats | Reproductive and Developmental Toxicity | Up to 1,200 mg/kg body weight/day. | No maternal or developmental toxicity was observed at any dose tested. | Mogroside V did not show any effects on parental reproductive performance, feta development, or survival in rats. This suggests a favorable safety profile for use during pregnancy. | ^63^ |
| Luo Han extract (Mogrosides) | Male and Female Dogs | Safety profile of mogrosides | 3000mg/kg bw/day | - | No changes in survival or body weight gain were found. No clinically relevant changes were observed. | ^61^ |
| Monk fruit extract (MFE) | Human studies | 0.51 g daily of MFE | Doses tested were aimed at providing sweetness equivalent to a caloric sweetener and were well-tolerated. | - | Clinical trials have shown that MFE does not affect postprandial glucose or insulin levels. No severe adverse effects have been reported. The primary concern is the limited long-term safety data in large-scale human populations. | ^64^ |
